# Validation of online mindfulness-enhanced course for stress reduction in teachers

**DOI:** 10.3389/fpsyt.2023.1086142

**Published:** 2023-04-04

**Authors:** Qun Ye, Ying Huang, Xingcheng Ge, Xiaolan Song

**Affiliations:** ^1^School of Psychology, Zhejiang Normal University, Jinhua, Zhejiang, China; ^2^Intelligent Laboratory of Child and Adolescent Mental Health and Crisis Intervention of Zhejiang Province, Zhejiang Normal University, Jinhua, Zhejiang, China

**Keywords:** teacher, mindfulness, online course, stress, emotion

## Abstract

**Background:**

Mindfulness-based interventions have gained popularity as a means of reducing stress and increasing resilience among the preclinical population. The present study aimed to investigate the effects of an online mindfulness-enhanced course on stress reduction in teachers, especially since online learning and teaching have been frequently applied to respond to emergencies such as COVID-19-relevant school suspension.

**Methods:**

The study consisted of two phases. Phase 1 aimed to explore the relationship between teachers' perceived stress and mindfulness traits. In total of 6,252 teachers completed assessments of stress symptoms using the Chinese Perceived Stress Scale (CPSS) and occupational stress sources, as well as mindfulness using the Five Factor Mindfulness Questionnaire (FFMQ). Phase 2 aimed to examine the effectiveness of the online mindfulness-enhanced course. In total of 132 teachers were randomly assigned to either receive a 3-week online mindfulness course specifically designed for stress reduction and emotion regulation (*N* = 66) or a matched active control group (*N* = 66) and their pre-training and post-training self-reported states (e.g., perceived stress, mindfulness level, practice time) were measured.

**Results:**

The detection rate of Health Risk Stress (≥26 scores) was as high as 61.72%, and a negative association between the score of FFMQ and perceived stress level was found. Importantly, compared to the control group, the mindfulness training group showed a significant decrease in perceived stress and negative emotion, as well as an increase in understanding of the core mechanisms of mindfulness after training. Additionally, individual improvement in FFMQ scores was predicted by practice time.

**Conclusions:**

The study showed a high percentage of teachers experiencing stress, and the data supported the reliability and validity of the brief online mindfulness-enhanced course designed to reduce stress and regulate emotion for frontline teachers.

## Introduction

Everyone experiences stress to some degree and stress can be defined as any type of change that causes cognitive, behavioral, emotional, or psychological tension (or strain) for individuals (1) and organizations (2). Increasing evidence shows that stress plays a critical role in the emergence of mental illness with extensive socio-economic consequences (3). Studies around the world have highlighted global stress prevalence and costs, particularly during and in the aftermath of the current COVID-19 pandemic (4). Unfortunately, the teaching profession is known to be highly stressful and demanding worldwide (5), and frontline teachers often report experiencing high levels of stress and burnout, which can negatively impact their physical and mental wellbeing. High rates of teacher occupational stress have been documented globally, which may be exacerbated by the pandemic (6).

A growing body of evidence indicates that the outbreak of the pandemic brings unprecedented challenges and acts as a severe external stressor that poses a significant threat to public mental health. For instance, the rates of stress for teachers were significantly higher than pre-COVID-19 rates across the world (7), thus the turnover rate for teachers more than doubled under the lockdown conditions. Furthermore, COVID-19 continued to increase teacher stress and burnout a year into the pandemic, with 72% of teachers feeling very or extremely stressed in the US (6). Therefore, there is an urgent need to provide teachers with appropriate interventions to prevent persistent or emerging long-term negative outcomes and to promote mental health (8, 9).

Mindfulness-based interventions (MBIs, e.g., mindfulness-based stress reduction), pioneered by Kabat-Zinn (10), have emerged as a promising approach to preventing mental health problems, with a central focus on regulatory mechanisms that enable more effective coping and stress reduction. Based initially on ancient contemplative traditions and informed by the principles of positive psychology, modern mindfulness-based interventions comprise a series of practices that bring awareness to present-moment experiences without judgment (11, 12). The beneficial effects of mindfulness-based interventions have been well documented, which include not only reducing symptoms of depression and anxiety in clinical populations (13) but also increasing the ability to be attentive and aware of the present moment in non-clinical populations (14, 15). Over the years, mindfulness-based interventions may be especially beneficial in populations exposed to high levels of stress (9).

However, traditional face-to-face mindfulness-based interventions (MBIs) are unlikely to be effective in the screen age. Traditional face-to-face MBIs are time-consuming and costly, and qualified instructors are relatively scarce (16), while alternative online mindfulness interventions can overcome the space-time limitations to some extent (17). Traditional face-to-face MBIs have been demonstrated to be effective in preventing the relapse of depression (18) and in reducing psychological distress and improving wellbeing in the non-clinical group (19), however, these MBIs would be limited by the reach and stigma (20).

Online MBIs, as a means of mindfulness-based self-help (MBSH) interventions, have received increasing attention in recent research to expand the potential availability of mindfulness training (66). Taylor et al. (66) conducted a meta-analysis of 83 studies to compare MBSH to control conditions on negative and positive emotion outcomes. The results showed small but statistically significant effects following the MBSH intervention. In addition, a recent new MBSH study (Internet-based self-help Mindfulness Intervention for Emotional Distress) also found that it could be effective in improving mindfulness and reducing anxiety and depression in patients with emotional disorders by using the Internet (20). However, it should be noted that low engagement is one of the challenges of existing online mindfulness intervention products (21). In addition, the moderating role of mindfulness traits between problem-solving coping styles and perceived stress levels is one of the mechanisms inherent in mindfulness-based stress reduction (22). Further, the Monitor and Acceptance Theory (65) suggests that mindfulness training involves a constant monitoring of the present experience and a permissive, non-judgmental attitude of acceptance. Mindfulness is a way of approaching experience and groups high in mindfulness traits tend to be better able to maintain awareness and acceptance of the present experience, and thus to respond intelligently to the experience itself rather than habitually, which is more conducive to both emotional regulation and problem-solving, and therefore serves to reduce stress.

Taken together, these findings provide evidence for the effectiveness of online mindfulness-based interventions on emotion regulation, but relatively few studies have focused on the impact of mindfulness-based interventions on stress reduction among non-clinical, vulnerable populations. The purpose of this two-phase study was to develop an online mindfulness-enhanced course and to validate the efficacy of the course in reducing teacher stress. First, an online survey was administered to examine the relationship between perceived stress and dispositional mindfulness traits, controlling for individual differences in demographic information among teachers. Inspired by the core factors contributing to the effectiveness of mindfulness training and the theoretical underpinnings of Mindfulness-Based Stress Reduction (MBSR), we then validated a 3-week online mindfulness course and explored its mechanism in reducing teacher stress in a randomized controlled trial.

## Materials and methods

### Participants

A total of 6,446 participants from elementary and secondary schools completed the survey, of whom 194 were excluded due to missing data or failure to complete the survey on time in Phase 1. The participants' demographic characteristics in Phase 1 are shown in Table 1. Phase 2 consisted of 199 elementary and secondary school teachers, of which 67 teachers were excluded due to the following conditions: previous experience in mindfulness learning and practice or related psychological courses during the intervention, history of psychiatric or neurological disorders, etc. Then, we randomly divided the remaining 132 teachers into a mindfulness training group and a cognitive learning group. Due to the loss of some participants during the post-test, the final number of participants was 97, including 42 in the mindfulness training group (7 males and 35 females) and 55 in the cognitive learning group (6 males and 49 females). The demographic information of the participants in Phase 2 is shown in Table 2. This study involving human participants was reviewed and approved by the ethics committee of Zhejiang Normal University. All participants provided their informed consent to participate in the study.

**Table 1:** Demographic characteristics of the survey sample in Phase 1.

**Demographic variables**	**Group**	**Number**	**Percentage**
Gender	Male	1,683	26.92%
	Female	4,569	73.08%
Stage of teaching	Elementary school	3,386	54.16%
	Secondary school	1,738	27.80%
	General high school	867	13.87%
	Secondary vocational school	261	4.17%
Years of teaching	<10 years	2,073	33.16%
	10–20 years	1,990	31.83%
	20–30 years	1,713	27.40%
	>30 years	476	7.61%
Coordinator of class	Yes	2,562	40.98%
	No	3,690	59.02%
Region of school	Urban	4,194	67.08%
	Rural	2,058	32.92%
Type of school	Public	5,922	94.72%
	Private	330	5.28%

**Table 2:** Demographic characteristics of the participants in Phase 2.

**Demographic variables**	**Group**	**Mindfulness training group (*n* [%])**	**Cognitive learning group (*n* [%])**
Gender	Male	7 (16.7%)	6 (10.9%)
	Female	35 (83.3%)	49 (89.1%)
Age	18–29	9 (21.4%)	28 (50.9%)
	30–49	32 (76.2%)	23 (41.8%)
	50–59	1 (2.4%)	4 (7.3%)
Years of teaching	<10 years	19 (45.2%)	33 (60.0%)
	11–20 years	10 (23.8%)	11 (20.0%)
	20–30 years	12 (28.6%)	9 (16.4%)
	>30 years	1 (2.4%)	2 (3.6%)
Stage of teaching	Elementary school	28 (66.7%)	17 (30.9%)
	Secondary school	8 (19.0%)	26 (47.3%)
	General high school	4 (9.5%)	8 (14.5%)
	Secondary vocational school	2 (4.8%)	4 (7.3%)
Coordinator of class	Yes	18 (42.9%)	23 (41.8%)
	No	24 (57.1%)	32 (58.2%)

### Procedure

This study used an online survey to examine the relationship between perceived stress and dispositional mindfulness traits in teachers in Phase 1, and then validated a targeted 3-week online mindfulness stress reduction course for teachers in a randomized controlled trial in Phase 2.

**Figure 1:**
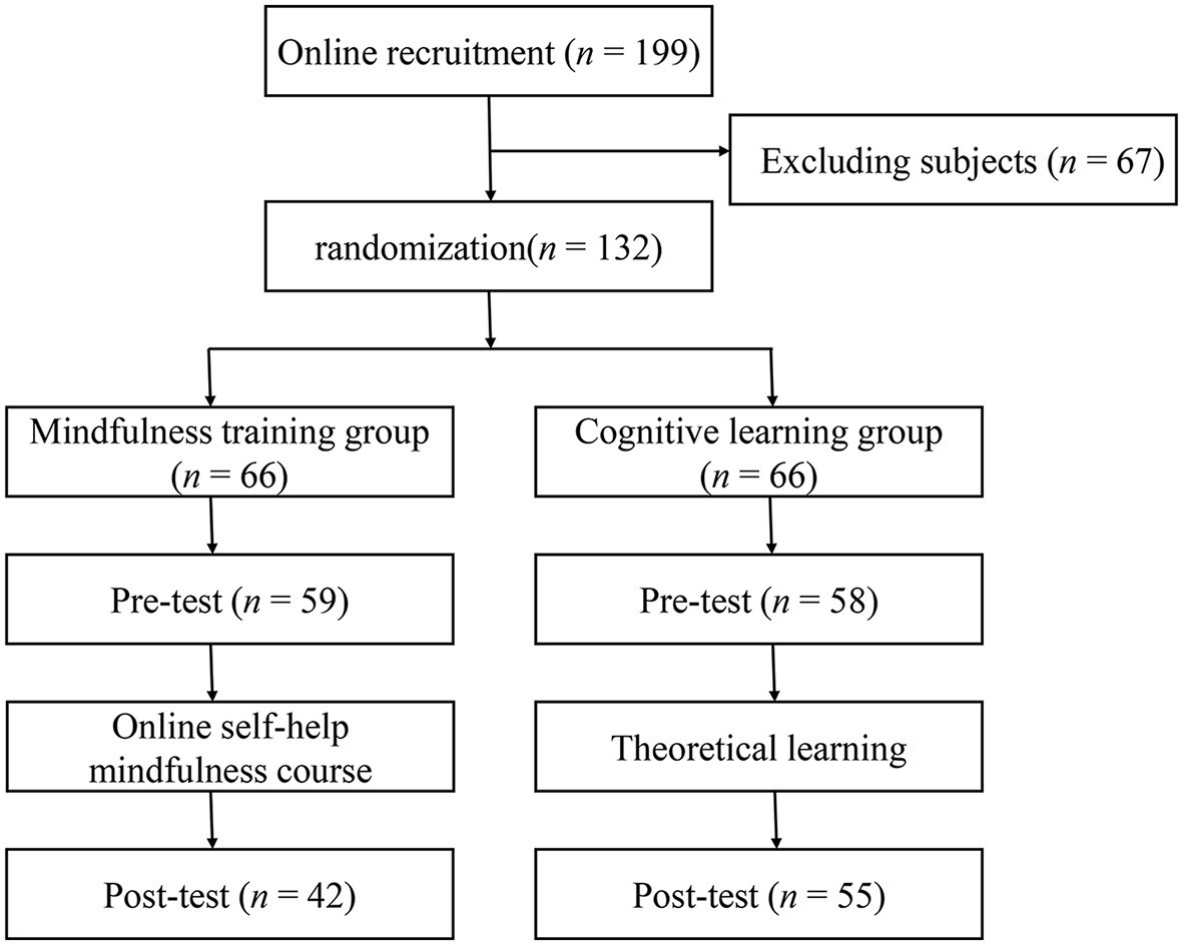
The flow chart of the study in Phase 2.

In Phase 1, participants were asked to complete a Chinese Perceived Stress Scale (CPSS), a Coping Style Scale, and a Five Facet Mindfulness Questionnaire (FFMQ). Through WeChat (Tencent Holdings Ltd.), we then established two groups (mindfulness training group vs. cognitive learning group) and published class announcements/instructions on how to use the online course and links to pretest scales in Phase 2. The mindfulness training group then participated in a 21-day online self-help mindfulness learning course, and the cognitive learning group participated in theoretical learning (including theoretical explanations of stress and mindfulness, the importance and benefits of participating in mindfulness training). Importantly, participants were required to record their feelings in a “Mindfulness APP” and the application would record the participants' learning time and days during the 21-day online training. Finally, both groups completed the post-test scale after the 21-day intervention. The flow chart of the study is shown in Figure 1. Besides, a Chinese Perceived Stress Scale (CPSS), a Short-Form Five Facet Mindfulness Questionnaire (SF-FFMQ), a Positive and Negative Affect Scale (PANAS), an Index of wellbeing (IWB) scale, a Chinese Big Five Personality Inventory brief version (CBF–PI–B) scale, a self-administered Cognition Scale of Mindfulness based Stress Reduction (CS-MBSR), and a self-administered Subjective Evaluation Questionnaire of the course were used in Phase 2.

Noted that we used an active control group, specifically a cognitive learning group, in our study to account for non-specific factors present in mindfulness training (24). The use of non-active control groups, such as waitlist controls, in randomized clinical trials of mindfulness interventions may result in specificity of outcomes (25). Active control groups, on the other hand, can provide a more accurate comparison between groups and help researchers learn more about the effectiveness of MBIs in relation to other evidence-based treatments (26). In our study, setting the cognitive learning group as the control group was done to exclude the influence of knowledge formed by “learning about mindfulness” and the influence of expectations such as “benefits of mindfulness” to some extent. This approach allows us to better assess the specific effects of the mindfulness intervention compared to an active control group.

### Online mindfulness-enhanced course

We developed a “Mindfulness App” as a learning platform for participants. The APP features one-click practice, automatic recording of practice data, and community sharing. Users can access the learning interface for practice with a single click to minimize resistance during the course. The app features high-quality audio guidance for practice, which is explained and guided by a senior mindfulness teacher with more than 300 h of teaching experience, and the audio is noise-reduced using professional software. In addition, elements such as practice recordings and statistics are used to fully motivate users. Users can record their practice experience in the recording box that pops up automatically after each practice and choose whether to share it or not. In the recording interface, users can see the times they have practiced, the total length of their practice, and a detailed record of their personal experience. In the statistics interface, the top, and bottom parts are the completion status of formal practice and daily practice, respectively. The gray icon would not change color until users had completed the day's practice, and the gray visual design provides intuitive feedback. Group dynamics and group guidance and support are important factors in facilitating users' mindfulness practice. Therefore, this APP incorporates a design that reflects group support, and users can see and interact with the shared experience of their peers' participation in the practice.

The content of the course was developed by the researcher and 6 senior MBSR teachers (more than 300 h of teaching experience) based on comprehensive research and discussion. The course lasted for 3 weeks and was developed in a progressive manner (Table 3). It takes about 15 min per day and consists of two parts that can be performed independently: formal exercise and daily practice. The formal exercises consist of two separate audios: the first is a short lecture on mindfulness, and the second is a guided audio for the formal exercises, both recorded by one senior MBSR teacher. The daily practices are a series of exercises that incorporate mindfulness techniques into daily activities. The specific content of the online mindfulness-enhanced course is shown in the Supplementary material. The whole course is divided into 3 weeks. The 1st week of the course is designed to help participants understand how mindfulness theory views the operation of mental processes such as attention, emotion and the basic intent of the course. The 2nd week is designed to help participants understand the principles of stress and the mechanisms of the mind-body response. Participants will learn how to live with stress using mindfulness awareness and will experience how mindfulness practice can alleviate the habitual response to stress. The 3rd week is designed to help participants use mindfulness techniques to deal with the ups and downs of everyday experiences in their lives, with an emphasis on introducing ways to respond to positive experiences in daily life that are easily overlooked.

**Table 3:** The general content of the online mindfulness-enhanced course.

**Week**	**Day**	**Formal practice (guided)**	**Daily practice (non-guided)**
1	1	Mindful writing	Focus on small things.
	2	Mindful breathing	Pay attention to breathing.
	3	Mindful eating	Mindful eating.
	4	Mindful breathing	Am I aware? Aware of distractions in daily life.
	5	Body scan (simplified version)	Pay attention to physical feelings in daily life.
	6	Body scan (simplified version)	Pay attention to physical feelings in daily life.
	7	Breathing and mindfulness of the body	Bedtime mindfulness
2	8	Self-care	Self-care in daily life
	9	Self-care	Self-care in daily life
	10	Get along with difficult emotions	Aware of body signals.
	11	Three-step breathing space (basic version)	Space in life: Pay attention, deliberately pause.
	12	Three-step breathing space (expansion version)	Using three-step breathing space in daily life.
	13	Three-step breathing space (basic version)	Space in life: Pay attention, deliberately pause.
	14	Body scan (full version)	Have a good sleep.
3	15	Mindful walking	Mindful walking in daily life.
	16	Mindfulness of breathing and body	Mindfulness practice at work
	17	Love-kindness	Mindfulness and good deeds.
	18	Love-kindness	Give thanks for the present.
	19	Mindful stretching	Give thanks for the present.
	20	Give thanks with ten fingers.	Mindful communication.
	21	sitting meditation	Mindful life plan.

Participants' lack of self-discipline was a major factor preventing them from participating in mindfulness practice. Therefore, the course assistants would send out a brief introduction of the day's course and a link to the program in the WeChat group early in the morning and a warm reminder in the evening to invite teachers who had not yet participated in the course to empower themselves through mindfulness practice after a hard day's work. Moreover, the course assistant and the lead teachers would provide timely feedback to help teachers better participate in the course by answering questions and resolving any confusion they encountered. As a control, the learning materials in the cognitive learning group included the theoretical explanations of stress and mindfulness, the importance and benefits of participating in mindfulness training. All the materials were short articles from related literature with the characteristics of science.

### Measures

#### Chinese perceived stress scale

The Perceived Stress Scale (PSS), developed by Cohen et al. (27), is a reliable scale to assess an individual's overall perception of stress. The original PSS has 14 items and is rated on a five-point Likert scale from 0 to 4, with higher scores indicating more significant stress. The Chinese version of the Perceived Stress Scale (CPSS) was revised by Yang and Huang (28), which has good reliability (Cronbach's α = 0.78).

#### Coping style scale

Xiao and Xu (29) developed this scale to measure individuals' strategies for coping with stressful events. The scale comprises 62 items with 6 dimensions (problem-focused, self-blame, help-seeking, image-distorting, avoidance, and rationalization). Each item is scored as 1 point for “Yes” and 0 for “No” except for 4 reverse scoring items. The Cronbach's α of the total scale in this study was 0.80, and the Cronbach's α of the six dimensions ranged from 0.57 to 0.83, suggesting acceptable reliability.

#### Five facet mindfulness questionnaire

Baer et al. (30) developed this scale to measure the level of mindfulness of individuals. The scale consists of 39 items with 5 dimensions (observe, describe, act with awareness, non-judging, and non-reactivity). In this study, the Cronbach's α of the total scale was 0.75, and the Cronbach's α of the five dimensions ranged from 0.67 to 0.86, suggesting good reliability.

#### Short-form five facet mindfulness questionnaire

Meng et al. (31) developed this scale. The SF-FFMQ has 20 items with 5 dimensions (observe, describe, act with awareness, non-judging, and non-reactivity). Each dimension has 4 items and all the items use a five-Likert scale from 1 to 5. In this study, the Cronbach's α of the total scale was 0.80 and the Cronbach's α of the five dimensions ranged from 0.75 to 0.90.

#### The positive and negative affect scale

Yang and Huang (28) developed the PANAS. This scale has 20 items and the response scale used a five-point Likert scale ranging from 1 to 5. Yang and Huang (28) conducted a study on the applicability of this scale in China and the results showed that the Cronbach's α for positive and negative affect were 0.85 and 0.83. In this study, the Cronbach's α for positive and negative affect in this study were 0.85 and 0.90.

#### Index of wellbeing

Campbell (32) developed the IWB. The IWB has 9 items including 8 items for Index of General Affect and 1 item for Life Satisfaction. The response scale uses a seven-point Likert scale ranging from 1 to 7. The total score is calculated by first reverse scoring, adding the mean score of Index of General Affect to the score of the Life Satisfaction scale^*^1.1, with the final score ranging between 2.1 (least happy) and 14.7 (happiest). In this study, the Cronbach's α was 0.94.

#### Chinese big five personality inventory brief version

Wang et al. (33) revised this scale. The CBF-PI-B has 40 items with 5 dimensions (extraversion, agreeableness, conscientiousness, neuroticism, and openness). The response scale uses a six-point Likert scale from 1 to 6. The Cronbach's α of the five dimensions ranged from 0.76 to 0.81. In this study, the Cronbach's α of the five dimensions ranged from 0.72 to 0.81. Note that the big five personality was used as a control variable in this study.

#### Cognition scale of mindfulness based stress reduction

The scale was self-administered and rated by five experts in Mindfulness Based Stress Reduction (MBSR). This scale reflects the respondents' understanding of the principles of mindfulness training and the mechanisms of stress formation. The scale has 8 items and includes 2 aspects. On the one hand, there are 6 items reflecting the core mechanism of mindfulness training. The 6 items are related to the 3 sub-dimensions of the IAA model of mindfulness training: intention, attention and attitude (34). On the other hand, there are 2 items about stress including two dimensions: the formation of stress and the relationship between stress and mindfulness. Each correct answer is counted as 1 point. The scale has good content validity (K^*^ > 0.74 for each item).

#### Subjective evaluation questionnaire on the course

This self-administered questionnaire has 4 items. The questions are “How did you like the course?”, “How interesting did you find this course?”, “To what extent are you willing to share this course with others for learning?” and “To what extent are you willing to explore and learn more about mindfulness?” The response scale uses a 4-point Likert scale from 1 to 4.

### Data analysis

In Phase 1, descriptive analysis was conducted on CPSS to understand the teacher's stress level. Then, correlational analysis was performed between CPSS and FFMQ to explore the relationship between perceived stress and mindfulness traits, and hierarchical regression analysis was used to analyze the effect of mindfulness traits on teacher's perceived stress after controlling the demographic variables. In Phase 2, independent samples *t-*tests were used to assess the differences between mindfulness training group and cognitive learning group. In addition, to test the effect of the mindfulness intervention, we used Big Five personality as a control variable and conducted a 2 (Group: mindfulness training vs. cognitive learning) × 2 (Time: pre-test vs. post-test) mixed-design analysis of variance (ANOVA) to detect differences in perceived stress, PANAS, wellbeing, mindfulness traits and CS-MBSR. Finally, to explore the relationship between the time teachers in the mindfulness training group spent in the practice and the effect of the course intervention, two partial correlation analysis (with the Big Five personality as control variables) were conducted between theoretical learning time, formal practice time and the change value of psychological and cognitive intervention effects.

## Results

### Mindfulness traits and perceived stress levels in teachers (Phase 1)

The scores on the CPSS scale were used to reflect the levels of psychological stress. A CPSS score of greater than or equal to 26 was used to designate an individual as Health Risk Stress (HRS) subject. A score of 42 was further considered as the cutoff point for more severe stress in this study. We found that 61.72% of the surveyed teachers had HRS (*M* = 27.54, *SD* = 7.97, 38.28% scored <26, 57.31% scored between 26 to 41, 4.41% scored ≥ 42).

We then analyzed the relationship between teachers' stress and mindfulness, and results showed a significant negative correlation between mindfulness and perceived stress levels (*r* = −0.506, *p* < 0.01). Furthermore, we conducted a hierarchical regression to analyze the relationship between demographic variables, mindfulness traits and perceived stress levels. The results showed that mindfulness significantly predicted perceived stress levels after controlling for these demographic variables (β = −0.504, *p* < 0.001, Table 4).

**Table 4:** Hierarchical regression for demographic variables and mindfulness trait predicting perceived stress level.

**Predictors**	**β**	**R^2^**	**ΔR^2^**	***F***
Gender		<0.001	<0.001	1.88
male	−0.035^**^			
Years of teaching		0.013	0.013	20.65^***^
<10 years	0.117^***^			
10–20 years	0.099^***^			
20–30 years	0.097^***^			
Teaching object		0.015	0.002	13.48^***^
elementary school	0.064^*^			
secondary school	0.056^*^			
general high school	0.071^***^			
Coordinator of class		0.020	0.005	15.52^***^
Yes	0.058^***^			
Type of school		0.021	0.001	14.67^***^
Public	0.018			
Region of school		0.021	0.000	13.25^***^
Urban	0.004			
Type of course		0.022	0.001	12.89^***^
main course	−0.006			
Mindfulness	−0.503^***^	0.268	0.246	190.63^***^

βs = standardized regression coefficients. R^2^ represents the percentage of the variance of the dependent variable that can be explained by the regression equation. ΔR^2^ represents the change in R^2^ attributable to the addition predictors to the model. ^*^p < 0.05, ^**^p < 0.01, ^***^p < 0.001.

### Online mindfulness-enhanced course for stress reduction in teachers (Phase 2)

First, to ensure that participants in the two groups remained the same at baseline, independent sample *t-*tests were used to examine the differences between mindfulness training group and cognitive learning group. The baseline psychometric indicators were analyzed using the sum score of subjective ratings that have been measured prior to the test. As is shown in Table 5, no significant between-groups differences were found for the baseline measurement of subjective ratings, including the perceived stress, PANAS, index of wellbeing, SF-FFMQ and CS-MBSR (all *p*s > 0.3). These results suggest that the subjective evaluation of these measures was comparable between groups.

**Table 5:** Baseline tests of psychometric indicators.

**Dimension**	***t***	***p***	**Mindfulness training group (*****M*** ±***SD*****)**		**Cognitive training group (*****M*** ±***SD*****)**	
			**Pre-test**	**Post-test**	**Pre-test**	**Post-test**
Perceived stress	−0.833	0.407	23.48 ± 8.36	19.43 ± 7.06	24.91 ± 8.44	25.24 ± 7.58
Positive affect	−0.124	0.902	29.83 ± 5.08	33.69 ± 4.98	29.96 ± 5.21	30.44 ± 5.24
Negative affect	−0.701	0.485	21.93 ± 7.26	20.05 ± 5.58	22.91 ± 6.21	24.31 ± 5.60
Index of wellbeing	0.003	0.998	9.69 ± 2.33	10.86 ± 2.55	9.69 ± 2.51	10.17 ± 2.14
Mindfulness trait	−0.591	0.556	60.69 ± 7.89	67.95 ± 7.61	61.67 ± 8.38	62.24 ± 7.17
Neuroticism	−0.26	0.795	26.10 ± 7.53		26.49 ± 7.32	
Conscientiousness	1.78	0.078	36.00 ± 6.03		33.84 ± 5.84	
Agreeableness	0.79	0.432	36.88 ± 4.75		36.07 ± 5.17	
Openness	−0.56	0.574	29.74 ± 6.31		30.49 ± 6.67	
Extraversion	−2.60	0.011	25.57 ± 5.44		28.27 ± 4.77	
Core mechanisms-overall score of CS-MBSR	0.735	0.464	3.64 ± 1.59	5.05 ± 1.36	3.40 ± 1.64	4.05 ± 1.72
Core mechanisms-intention of CS-MBSR	0.048	0.962	1.26 ± 0.73	1.62 ± 0.62	1.25 ± 0.75	1.42 ± 0.71
Core mechanisms-attention of CS-MBSR	0.671	0.504	1.07 ± 0.56	1.64 ± 0.62	0.98 ± 0.76	1.13 ± 0.75
Core mechanisms-attitude of CS-MBSR	0.903	0.369	1.31 ± 0.84	1.79 ± 0.61	1.16 ± 0.71	1.51 ± 0.72
Stress-overall score of CS-MBSR	0.232	0.817	1.38 ± 0.76	1.62 ± 0.66	1.35 ± 0.73	1.49 ± 0.63
Stress-formation of the stress of CS-MBSR	−0.020	0.984	0.76 ± 0.43	0.90 ± 0.30	0.76 ± 0.43	0.87 ± 0.34
Stress-relationship between stress and mindfulness of CS-MBSR	0.368	0.714	0.62 ± 0.49	0.71 ± 0.46	0.58 ± 0.50	0.62 ± 0.49

Next, to examine the effect of the psychological and cognitive intervention of the course, we used Big Five personality as a control variable and conducted a 2 (Group: mindfulness training vs. cognitive learning) × 2 (Time: pre-test vs. post-test) mixed-design analysis of variance (ANOVA).

#### Perceived stress

**Figure 2:**
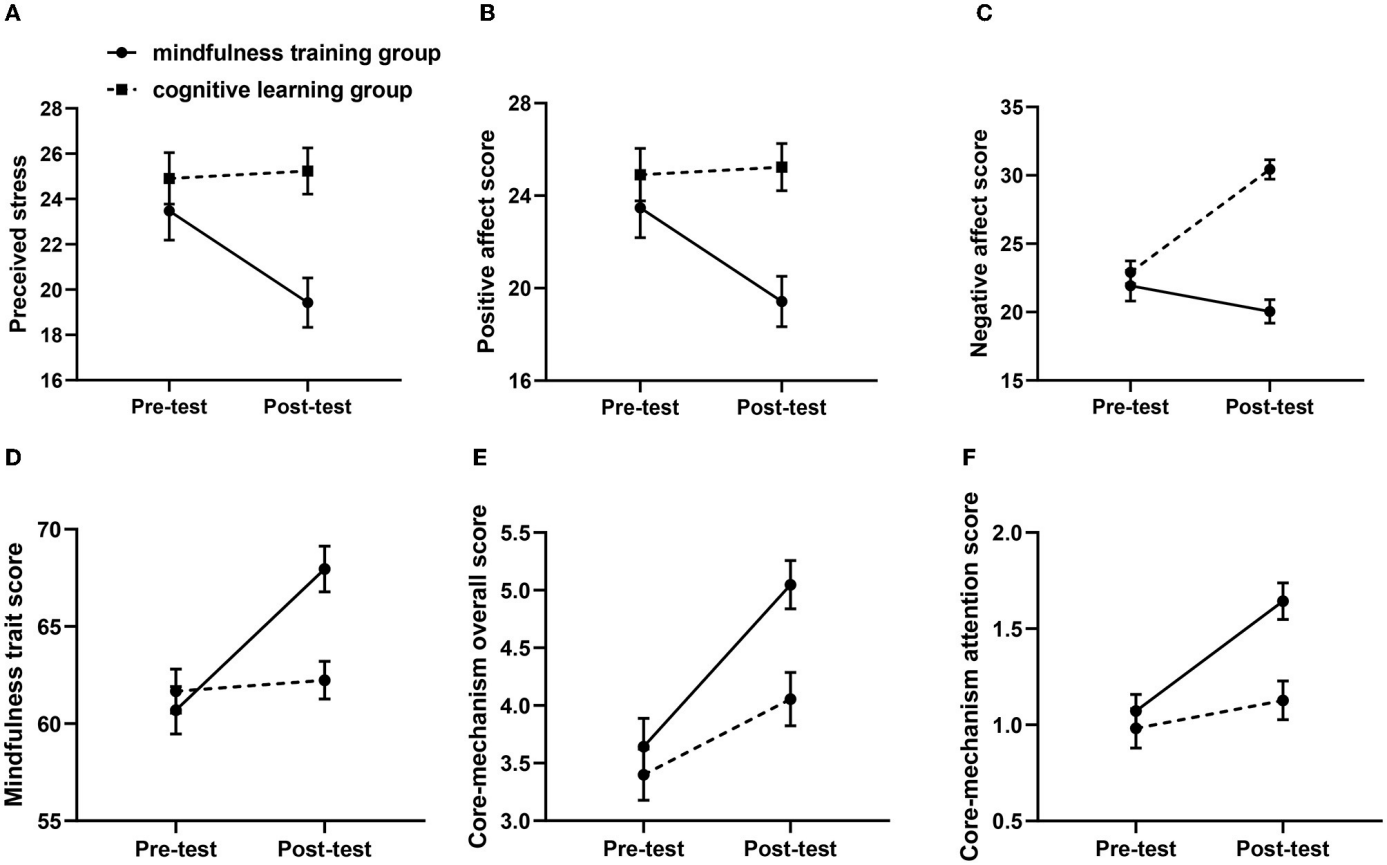
**(A–F)** The effect of the psychological and cognitive intervention of the course in Phase 2.

The mixed-design ANOVA yielded a significant main effect of group, *F*_(1, 95)_ =7.664, *p* = 0.007, *η_p_*^2^ = 0.078, a non-significant main effect of time, *F*_(1, 95)_ = 1.801, *p* = 0.183, *η_p_*^2^ = 0.020, and a significant time and group interaction (Figure 2A), *F*_(1, 95)_ = 7.628, *p* = 0.007, *η_p_*^2^ = 0.078. Follow-up simple effects analysis revealed that the level of perceived stress was significantly decreased after the intervention in the mindfulness training group, *F*_(1, 95)_ = 17.665, *p* < 0.001, *η_p_*^2^ = 0.164, while there was no significant difference between the pre-test and the post-test in the cognitive learning group, *F*_(1, 95)_ = 0.110, *p* = 0.741, *η_p_*^2^ = 0.001.

#### PANAS

For positive affect, the results showed main effects of both group, *F*_(1, 95)_ = 3.943, *p* = 0.050, *η_p_*^2^ = 0.042, and time, *F*_(1, 95)_ = 6.292, *p* = 0.014, *η_p_*^2^ = 0.065. The Group × Time interaction was significant (Figure 2B), *F*_(1, 95)_ = 10.997, *p* = 0.001, *η_p_*^2^ = 0.109. The scores of positive affect were significantly higher on the post-test than on the pre-test in the mindfulness training group, *F*_(1, 95)_ = 27.241, *p* < 0.001, *η_p_*^2^ = 0.232, but no significant difference in the cognitive learning group, *F*_(1, 95)_ = 0.645, *p* = 0.424, $\eta_{\text{p}}^{2}$ = 0.007, indicating that mindfulness training was fully effective for increasing the positive affect. For negative affect, a significant main effect of group was found, *F*_(1, 95)_ = 4.123, *p* = 0.045, *η_p_*^2^ = 0.044, but no difference of time, *F*_(1, 95)_ = 0.165, *p* = 0.686, *η_p_*^2^ = 0.002. The interaction was significant (Figure 2C), *F*_(1, 95)_ = 8.455, *p* = 0.005, *η_p_*^2^ = 0.086. Planned comparisons revealed that participants in the mindfulness training group decreased negative affect significantly after the intervention, *F*_(1, 95)_ = 27.241, *p* < 0.001, *η_p_*^2^ = 0.232, but no difference in the cognitive learning group, *F*_(1, 95)_ = 0.645, *p* = 0.424, *η_p_*^2^ = 0.007.

#### Index of wellbeing

The mixed-design ANOVA results showed non-significant main effects of both group, *F*_(1, 95)_ = 0.00, *p* = 0.990, *η_p_*^2^ = 0.000, and time, *F*_(1, 95)_ = 0.001, *p* = 0.973, *η_p_*^2^ = 0.000, as well as a non-significant interaction effect, *F*_(1, 95)_ = 2.620, *p* = 0.109, *η_p_*^2^ = 0.028.

#### Mindfulness trait

A mixed-design ANOVA returned that no differences were found for both group, *F*_(1, 95)_ = 1.547, *p* = 0.217, *η_p_*^2^ = 0.017, and time, *F*_(1, 95)_ = 1.972, *p* = 0.164, *η_p_*^2^ = 0.021. However, the interaction between time and group was significant as expected (Figure 2D), *F*_(1, 95)_ = 22.879, *p* < 0.001, *η_p_*^2^ = 0.203. In the mindfulness training group, a significant difference of time emerged, *F*_(1, 95)_ = 46.985, *p* < 0.001, *η_p_*^2^ = 0.343, whereas in the cognitive learning group, no significant difference, *F*_(1, 95)_ = 0.145, *p* = 0.704, *η_p_*^2^ = 0.002, indicating that teachers in the mindfulness training group had significant change of mindfulness traits.

#### Core-mechanism of CS-MBSR

For this dimension, there were three sub-dimensions: attention, intention and attitude. First, the overall score was analyzed. The mixed-design ANOVA yield a marginal significant main effect of group, *F*_(1, 95)_ = 3.197, *p* = 0.077, *η_p_*^2^ = 0.034, but no difference of time, *F*_(1, 95)_ = 0.501, *p* = 0.481 *η_p_*^2^ = 0.006. The group × time interaction was significant (Figure 2E), *F*_(1, 95)_ = 6.445, *p* = 0.013, *η_p_*^2^ = 0.067. The scores of mindfulness training group were significantly higher than the cognitive learning group in the post-test, *F*_(1, 95)_ = 7.850, *p* = 0.006, *η_p_*^2^ = 0.080, but no difference in the pre-test, *F*_(1, 95)_ = 0.097, *p* = 0.756, *η_p_*^2^ = 0.001. Thus, self-help mindfulness course made participants higher cognitive understanding of the core mechanisms of mindfulness training. Then, the same results were found in the dimension of attention [Group: *F*_(1, 95)_ = 6.093, *p* = 0.015, *η_p_*^2^ = 0.063; Time: *F*_(1, 95)_ = 0.622, *p* = 0.432, *η_p_*^2^ = 0.007; Group × Time interaction (Figure 2F): *F*_(1, 95)_ = 5.889, *p* = 0.017, *η_p_*^2^ = 0.061; in post-test, *F*_(1, 95)_ =11.314, *p* = 0.001, *η_p_*^2^ = 0.112; in pre-test, *F*_(1, 95)_ = 0.431, *p* = 0.513, *η_p_*^2^ = 0.005]. However, no differences were found for both attitude [Group: *F*_(1, 95)_ = 0.579, *p* = 0.449, *η_p_*^2^ = 0.006; Time: *F*_(1, 95)_ = 0.721, *p* = 0.398, *η_p_*^2^ = 0.008; Group × Time interaction: *F*_(1, 95)_ = 2.028, *p* = 0.158, *η_p_*^2^ = 0.022] and intention [Group: *F*_(1, 95)_ = 0.651, *p* = 0.422, *η_p_*^2^ = 0.007; Time, *F*_(1, 95)_ = 1.393, *p* = 0.241, *η_p_*^2^ = 0.015; Group × Time interaction: *F*_(1, 95)_ = 2.138, *p* = 0.147, *η_p_*^2^ = 0.023].

Then, to explore the relationship between the time teachers in the mindfulness training group spent in the practice and the effect of the course intervention, two partial correlation analysis (with the Big Five personality as control variables) were conducted between theoretical learning time (*M* = 102.60 min, *SD* = 31.61), formal practice time (*M* = 332.12 min, *SD* = 158.94) and the change value of psychological (Table 6) and cognitive intervention effects (Table 7). The results showed that total time of practice was significantly and positively correlated with the value of change in mindfulness traits (*r* = 0.363, *p* = 0.027), theoretical learning time was marginal significantly and positively correlated with the value of change in mindfulness traits (*r* = 0.289, *p* = 0.083), and formal practice time was significantly and positively correlated with the value of change in mindfulness traits (*r* = 0.356, *p* = 0.030). It was necessary to add that we counted the actual number of days teachers participated in the course and found that all teachers in the mindfulness training group participated for 11 days or more, 83.33% participated for 15 days or more, and 35.71% participated for 21 days.

**Table 6:** Partial correlation between exercise time and change in psychological intervention effect values.

	**1**	**2**	**3**	**4**	**5**	**6**	**7**
1. Total time of practice	—						
2. Theoretical learning time	0.743^***^	—					
3. Formal practice time	0.991^***^	0.650^***^	—				
4. ΔPerceived stress level	−0.205	−0.164	−0.201	—			
5. ΔPositive affect	0.067	0.241	0.029	−0.596^***^	—		
6. ΔNegative affect	−0.194	−0.201	−0.182	0.818^***^	−0.492^**^	—	
7. ΔMindfulness trait	0.363^*^	0.289^*^	0.356^*^	−0.699^***^	0.513^**^	−0.618^***^	—

^*^p < 0.05, ^**^p < 0.01, ^***^p < 0.001. The Δperceived stress level is the difference between the post-test perceived stress level and the pre-test perceived stress level, and all other Δ measures are the differences between the post-test score and the pre-test score. Same below.

**Table 7:** Partial correlation between exercise time and change in cognitive intervention effect values.

	**1**	**2**	**3**	**4**	**5**
1. Total time of practice	—				
2. Theoretical learning time	0.743^***^	—			
3. Formal practice time	0.991^***^	0.650^***^	—		
4. ΔCore Mechanisms-overall score	−0.072	−0.050	−0.072	—	
5. ΔCore Mechanisms-attention	−0.116	−0.090	−0.114	0.577^***^	—

^*^p <0.05, ^**^p <0.01, ^***^p <0.001.

Finally, we found that teachers in the mindfulness training group showed overall good subjective evaluations of the course. The mean scores for the degree of willingness to share the course with others (*M* = 3.43, *SD* = 0.70) and the degree of willingness to further explore and learn about mindfulness (*M* = 3.29, *SD* = 0.74) in the mindfulness training group were higher than the mean value, reflecting the teachers' positive willingness to share this course and to continue practicing mindfulness.

## Discussion

The aim of this study was to investigate the effectiveness of an online mindfulness-enhanced course in reducing stress and enhancing cognitive wellbeing among teachers. Overall, results indicated a high rate of health risk stress in teachers, which highlights the need for effective stress management interventions in the education sector. However, the completion of the 3-week online course was associated with a significant reduction in perceived stress levels and significant improvements in mindfulness traits, positive affect, and cognitive understanding of the core mechanisms of mindfulness training among participants. These findings are consistent with previous studies that suggest mindfulness interventions can improve various aspects of wellbeing. Therefore, the results of this study have important implications for the future application of mindfulness-based interventions in educational settings.

The prevalence of Health Risk Stress among teachers was higher than the standard level of urban residents in China, indicating a need for effective stress-reducing interventions (28). Mindfulness, as a personal trait, has been shown to have a negative association with perceived stress (35), with some studies suggesting that it mediates the relationship between problem-solving coping styles and perceived stress levels (22). The underlying mechanism of mindfulness-based stress reduction (MBSR), which emphasizes the cultivation of a non-reactive and non-judgmental attitude toward present experiences, provides some evidence for the hypothesis that the course may be effective in reducing teachers' stress (36, 67). As expected, the course customized for teachers was proven to be feasible and efficacious in reducing teachers' perceived stress and negative affect and improving positive affect, which was consistent with previous studies (37–41, 67). For instance, Jennings et al. (40) found that a mindfulness-based intervention (MBI) effectively reduced stress and burnout. A theoretical premise of MBI is that developing of mindfulness skills leads to a non-judgmental and non-reactive acceptance of all experiences (42, 43). Acceptance may help individuals regulate their stress reactivity by facilitating the recognition and subsequent disengagement from all momentary sensory experiences and stressful difficulties (44), thus improving psychological flexibility (45).

Attention dimension of our scale was significantly increased after course intervention but no differences in intention and attitude. Indeed, improvement in attention has been observed in individuals who participate in MBSR programs (46, 47). Furthermore, a study conducted by MacLean et al. (48) found that regulating attention through mindful breathing practices resulted in a significant improvement in attentional performance over 3 months of training, demonstrating the practical benefits of mindfulness training. It is worth noting that attention is a fundamental component of mindfulness (49). The mindfulness training in our study required participants to observe their internal and external experiences, which allowed for the practice and improvement of attentional abilities. Although we observed a significant improvement in attention, no significant differences were found in the intention and attitude dimensions of our scale. It is possible that the control group's learning experience, which included theoretical knowledge related to the benefits and precautions of mindfulness training, may have influenced their intention and attitude toward mindfulness, thus reducing the differences between the two groups.

The present study also investigated the effect of mindfulness training on the wellbeing of teachers. However, no significant differences were found in the wellbeing of participants in the two groups. While some studies have reported no significant effects of mindfulness training on wellbeing (36), several others have demonstrated that mindfulness interventions can lead to improvements in wellbeing and life satisfaction. Subjective wellbeing has been conceptualized as comprising both trait and state components (50). The trait component, which is relatively stable and influenced by individual genetic and personality traits (51), includes general affect. On the other hand, the state component, which is more malleable and can be influenced by contextual factors, includes life satisfaction (32). Given that the measurement scale adopted in Phase 2 of the present study only included one item for life satisfaction, it may not have been adequate to capture changes in the state component of wellbeing (52). Therefore, future research should consider using a multi-item measurement scale, such as the Satisfaction With Life Scale (SWLE) (53), to assess subjective wellbeing in a more comprehensive manner. It is worth noting that mindfulness training was originally developed for clinical populations, who typically have a strong desire for rehabilitation and high expectations for intervention (54). In contrast, the present study focused on non-clinical populations, specifically teachers. As such, it is possible that the relatively stable nature of teachers' subjective wellbeing may have limited the impact of the mindfulness intervention. Nevertheless, given the potential benefits of mindfulness training for individuals in non-clinical settings, further research is warranted to explore the factors that may moderate the effects of mindfulness interventions on wellbeing in this population.

A self-service online mindfulness course of 3 weeks' duration was implemented in our study, which differed from traditional mindfulness-based interventions such as MBCT and MBSR. The optimal amount of time required for effective mindfulness practice has been a topic of debate in the literature. Some researchers have argued that a longer practice time results in a greater level of expertise and higher quality of the mindfulness experience, thereby leading to better intervention outcomes (55). In contrast, others have found that shorter mindfulness training sessions, lasting <8 weeks, may be more effective (23, 56). Our study involved a 3-week short-term mindfulness training course with a relatively high training intensity, requiring participants to engage in daily mindfulness practice. The current findings, in line with the debate surrounding practice time, suggest that the quality of mindfulness practice may be more important than the duration of practice in achieving desired outcomes (57). Attitude, a key element of mindfulness in IAA theory (34), is associated with the quality of attention. Without the proper attitudinal qualities, the practice may become critical or judgmental of inner or outer experiences (58). Therefore, it is crucial to strike a balance between the duration of practice and the quality of practice to achieve the desired outcomes.

The present study has some limitations that require further consideration. First, although the results of the study suggest that online mindfulness-enhanced stress reduction may be an effective mechanism for change, some unmeasured factors may have influenced the intervention. Specifically, previous studies have reported that teachers' self-efficacy can reduce stress and general psychological distress (59, 60). Future studies could incorporate these related factors to comprehensively assess the effectiveness of mindfulness interventions in teachers. Second, the subjective nature of self-report data may have been influenced by various factors, such as emergent events, which could have compromised the objectivity and accuracy of the data. To provide a more comprehensive and objective assessment of mindfulness interventions, future studies could employ physiological data. For instance, research in neuroscience has demonstrated that mindfulness practices can influence neuroplasticity in brain regions associated with attention control, emotion regulation, and self-awareness (49, 61–63).

## Implications of the study

The online self-help mindfulness-enhanced course designed in this study significantly reduced teachers' perceived stress and negative emotions and improved their positive emotions, mindfulness traits and understanding of the core mechanism of mindfulness training during the 21-day short-term training. Although the effectiveness of online mindfulness intervention has been demonstrated in many researches (64), the present study still has some unique value.

First, the course was tailored to the characteristics of the audience, focusing on teachers' stress reduction and designed to fit into their daily routines. The 21-day course is divided into short, easily manageable modules, allowing participants to learn and practice mindfulness principles at their own pace. Second, the course design is rooted in mindfulness learning principles and user experience, making it distinct from other online mindfulness courses and apps. In terms of practice amount, the course strikes a balance between feasibility and practice time. The course content is designed with the perspective of a “teacher” teaching mindfulness and a “student” using the product in mind, rather than focusing solely on “fast-food mindfulness” or a “mindfulness course as a practice audio package.” Third, the course clearly demonstrates the fundamental orientation of mindfulness training toward daily life. Formal practice and daily practice, like the two wings of mindfulness training, are emphasized in all MBSR courses, including this one.

Generally speaking, the current research designed and verified an online mindfulness-enhanced reduction course, which can effectively reduce the perceived stress and negative emotions of primary and secondary school teachers, while improving their positive emotions and mindfulness traits. This course was helpful to the physical and mental health of teachers to some extent, which was one of the influencing factors of the quality of teaching, so the research on the relationship between the course and the teaching quality can be conducted to explore applications.

## Data availability statement

The raw data supporting the conclusions of this article will be made available by the authors, without undue reservation.

## Ethics statement

The studies involving human participants were reviewed and approved by the Ethics Committee of Zhejiang Normal University. The patients/participants provided their written informed consent to participate in this study.

## Author contributions

XS and XG designed the research. QY, YH, XG, and XS collected the data and performed the statistical analysis. QY, YH, and XS wrote the first draft of the manuscript. All authors contributed to the research and approved the final version of the manuscript.
