# Supplementary material for: Validation of online mindfulness-enhanced course for stress reduction in teachers
Source: Front Psychiatry. 2023 Apr 4;14:1086142. doi: 10.3389/fpsyt.2023.1086142 (PMC10110849; doi:10.3389/fpsyt.2023.1086142)
Supplement: Supplementary file 1 [file Table_1.DOCX]

Supplementary Material

# Supplementary Table

**Supplementary Table 1 |** The specific content of the Online Mindfulness-enhanced Course

| Week | Day | Course Topic | Theoretical lecture | Formal practice | Daily practice |
| --- | --- | --- | --- | --- | --- |
| 1 | 1 | Mindfulness for Sailing | Focused and joyful happiness. | Mindful writing (aim to Concentration of mind and build connection with body) | Focus on small things. |
|  | 2 | Mindfulness from ancient times to the present | Basis of contemporary mindfulness: wisdom spanning thousands of years meets the modern psychology. | Mindful breathing: ancient people and modern people are breathing. | Pay attention to breathing. |
|  | 3 | Living in the present moment | Pay attention to the present: recognize the physical and mental experience. | Mindful eating | Concentrate on a meal. |
|  | 4 | How to pay attention properly | Awareness: Be your own master. | Mindful breathing: wisdom to deal with distractions | Am I aware? Aware of distractions in daily life. |
|  | 5 | Awakening the busy mind | The art of balancing Process and Goal | Body Scan (Simplified version) | Pay attention to physical feelings in daily life. |
|  | 6 | Rest of heart, true rest | Efficient rest: what happened during the body scan? | Body Scan (Simplified version) | Pay attention to physical feelings in daily life. |
|  | 7 | Wholeheartedness, mind and body together | Seven attitudes of Mindfulness | Breathing and Mindfulness of the Body | Bedtime peaceful ritual |
| 2 | 8 | Stress and me | My career and stress | Self-care | Self-care in daily life |
|  | 9 | Understanding stress | What happens to the body under stress? | Self-care | Self-care in daily life |
|  | 10 | Transforming the inertia reaction of stress | The magical power of Permission. | Get along with difficult emotions | Aware of body signals. |
|  | 11 | Responding to stress with wisdom | Create space and go beyond inertia reaction. | Three-step breathing space (basic version) | Space in life: pay attention, deliberately pause. |
|  | 12 | True space – deepening breathing space | Deepen breathing space. | Three-step breathing space (expansion version) | Using three-step breathing space in daily life. |
|  | 13 | Why mindfulness needs practice1 | Get out of autopilot | Three-step breathing space (basic version) | Space in life: pay attention, deliberately pause. |
|  | 14 | Why mindfulness needs practice 2 | Psychology and Neuroscience of stress reaction | Body scan (full version) | Have a good sleep. |
| 3 | 15 | Unbiased attention | Unbiased attention | Mindfulness walking | Mindful walking in daily life. |
|  | 16 | Mindfulness is the foundation of self-care | Self-care at work | Breathing and mindfulness of the body | Mindfulness practice at work |
|  | 17 | Kindness from the inside out | Kindness from the inside out | Love-kindness | Mindfulness and good deeds. |
|  | 18 | True Care | Candles and Gardeners | Love-kindness | Give thanks to the present. |
|  | 19 | The Good in life | Restore the beauty of life with mindfulness | Mindful stretching | Give thanks to the present. |
|  | 20 | Bringing mindfulness to interpersonal interaction | How to use Mindfulness in interpersonal interaction. | Give thanks with ten fingers. | Mindful communication. |
|  | 21 | 21 Days to Begin and End | Let mindfulness become a way of life. | sitting meditation | plan your life with mindfulness |
